# Molecular characterization of common zoonotic protozoan parasites and bacteria causing diarrhea in dairy calves in Ningxia Hui Autonomous Region, China[Fn FN1]

**DOI:** 10.1051/parasite/2024059

**Published:** 2024-10-01

**Authors:** Jia-Qi Zhao, Ying-Ying Fan, Yun-Duan Lei, Ding Liu, Jun-Wei Wang, Xin Yang, Jun-Ke Song, Guang-Hui Zhao

**Affiliations:** College of Veterinary Medicine, Northwest A&F University Yangling 712100 PR China

**Keywords:** *Cryptosporidium* spp., *Giardia duodenalis*, *Enterocytozoon bieneusi*, *Escherichia coli*, *Clostridium perfringens*, *Salmonella* spp.

## Abstract

Diarrhea caused by zoonotic pathogens is one of the most common diseases in dairy calves, threatening the health of young animals. Humans are also at risk, in particular children. To explore the pathogens causing diarrhea in dairy calves, the present study applied PCR-based sequencing tools to investigate the occurrence and molecular characteristics of three parasites (*Cryptosporidium* spp., *Giardia duodenalis*, and *Enterocytozoon bieneusi*) and three bacterial pathogens (*Escherichia coli*, *Clostridium perfringens*, and *Salmonella* spp.) in 343 fecal samples of diarrheic dairy calves from five farms in Lingwu County, Ningxia Hui Autonomous Region, China. The total positive rate of these pathogens in diarrheic dairy calves was 91.0% (312/343; 95% CI, 87.9–94.0), with *C. perfringens* (61.5%, 211/343; 95% CI, 56.3–66.7) being the dominant one. Co-infection with two to five pathogens was found in 67.3% (231/343; 95% CI, 62.4–72.3) of investigated samples. There were significant differences (*p* < 0.05) in the positive rates of *Cryptosporidium* spp. and diarrheagenic *E. coli* among farms, age groups, and seasons. Two *Cryptosporidium* species (*C. parvum* and *C. bovis*) and five *gp60* subtypes of *C. parvum* (IIdA15G1, IIdA20G1, IIdA19G1, IIdA14G1, and a novel IIdA13G1) were identified. Two assemblages (assemblage E and zoonotic assemblage A) of *G. duodenalis* and six ITS genotypes of *E. bieneusi* (J, Henan-IV, EbpC, I, EbpA, and ESH-01) were observed. Four virulence genes (*eaeA*, *stx1*, *stx2*, and *st*) of diarrheagenic *E. coli* and one toxin type (type A) of *C. perfringens* were detected. Our study enriches our knowledge on the characteristics and zoonotic potential of diarrhea-related pathogens in dairy calves.

## Abstract (fr)

La diarrhée causée par des agents pathogènes zoonotiques est l’une des maladies les plus courantes chez les veaux laitiers, menaçant la santé des jeunes animaux. Ceci est également un risque pour la santé humaine, en particulier les enfants. Pour explorer les agents pathogènes responsables de la diarrhée chez les veaux laitiers, cette étude a utilisé des outils de séquençage basés sur la PCR pour étudier l’occurrence et les caractères moléculaires de trois parasites (*Cryptosporidium* spp., *Giardia duodenalis* et *Enterocytozoon bieneusi*) et de trois agents pathogènes bactériens (*Escherichia coli*, *Clostridium perfringens* et *Salmonella* spp.) dans 343 échantillons fécaux de veaux laitiers diarrhéiques provenant de cinq fermes du comté de Lingwu, région autonome Hui du Ningxia, en Chine. Le taux total positif de ces pathogènes chez les veaux laitiers diarrhéiques était de 91,0 % (312/343; IC à 95 %, 87,9–94,0), et *C. perfringens* (61,5 %, 211/343; IC à 95 %, 56,3–66,7) était le plus répandu. Une co-infection avec deux à cinq pathogènes a été trouvée dans 67,3 % (231/343; IC à 95 %, 62,4–72,3) des échantillons étudiés. Il y avait des différences significatives (*p* < 0,05) dans les taux positifs de *Cryptosporidium* spp. et d’*E. coli* diarrhéogènes entre les fermes, les groupes d’âge et les saisons. Deux espèces de *Cryptosporidium* (*C. parvum* et *C. bovis*) et cinq sous-types de *gp60* de *C. parvum* (IIdA15G1, IIdA20G1, IIdA19G1, IIdA14G1 et un nouveau, IIdA13G1) ont été identifiés. Deux assemblages (assemblage E et assemblage zoonotique A) de *G. duodenalis* et six génotypes ITS d’*E. bieneusi* (J, Henan-IV, EbpC, I, EbpA et ESH-01) ont été observés. Quatre gènes de virulence (*eaeA, stx1, stx2 et st*) d’*E. coli* diarrhéogènes et un type de toxine (type A) de *C. perfringens* ont été détectés. Notre étude enrichit les connaissances sur les caractères et le potentiel zoonotique des agents pathogènes liés à la diarrhée chez les veaux laitiers.

## Introduction

Diarrhea, the most common disease in cattle, has been reported as one of the leading causes of death in dairy calves. It has been identified as the highest factor responsible for morbidity and mortality events recorded in dairy calves [1], and occurs in dairy calves due to genetics, nutritional, and infectious factors [3]. Zoonotic pathogens including *Cryptosporidium* spp., *Giardia duodenalis*, *Enterocytozoon bieneusi*, *Escherichia coli*, *Clostridium perfringens*, and *Salmonella* spp. are generally considered to be the major enteric pathogens to cause cattle diarrhea [16, 20, 49, 60]. Cattle are commonly infected with four *Cryptosporidium* species, namely *C. parvum*, *C. bovis*, *C. ryanae*, and *C. andersoni* [69], and *C. parvum* has been reported to be the most important zoonotic species in humans and animals [23]. Three subtype families (Ⅱa, Ⅱd and Ⅱc) of *C. parvum* were detected in calves worldwide by using a subtyping tool targeting the *gp60* gene, and subtypes IIdA15G1 and IIdA19G1 have been found to be predominant in China [14]. Of eight assemblages (A–H) within *G. duodenalis*, assemblage E was dominant in cattle, followed by zoonotic assemblage A and mixed assemblages A and E by using molecular methods targeting *bg*, *gdh*, and *tpi* gene loci [59]. *Enterocytozoon bieneusi* ITS genotypes BEB4, I, and J in genetic Group 2 have previously been widely documented in cattle, but they have subsequently been found in humans and other animals, and exhibit zoonotic potential [40, 58]. Infection with diarrheagenic *E. coli* (DEC) leads to inflammation and diarrhea in infected cattle and humans [44]. *Clostridium perfringens* can cause a variety of diseases in calves and different hosts, including gas gangrene, enterotoxemia, and necrotizing enterocolitis [62]. *Salmonella* spp. are one of the most important foodborne and/or zoonotic pathogens that can infect a wide range of hosts [17]. Although several serotypes of *Salmonella* spp. can infect cattle, two main serotypes of bovine salmonellosis are *S. typhimurium* and *S. dublin* [48, 50]*.*

Cattle are important economic animals in China, providing high quality milk and meat for the population’s daily needs. Lingwu county is located in the golden milk belt, with the milk industry as an important pillar of economic development. Diarrhea remains the most common disease in calves on dairy farms and causes significant economic losses due to the complexity and diversity of infectious factors [67]. An increasing number of studies have reported that cattle are important reservoirs for *Cryptosporidium* spp., *G. duodenalis*, *E. bieneusi*, *E. coli*, *C. perfringens*, and *Salmonella* spp., leading to diarrhea in dairy calves [15, 36, 41, 53]. More importantly, there is concern about cross-species transmission of these zoonotic pathogens, which threatens the health of humans and other animals. However, knowledge on the colonization frequency of these pathogens is limited. This study investigated the occurrence of *Cryptosporidium* spp., *G. duodenalis*, *E. bieneusi*, *E. coli, C. perfringens*, and *Salmonella* spp. in dairy calves with diarrhea from Lingwu county, Ningxia Hui Autonomous Region, China, and the results could help us understand more deeply the role of these pathogens causing diarrhea in dairy calves and the zoonotic potential of these pathogens.

## Materials and methods

### Ethics statement

Before beginning this study, we described the protocol to the farm managers and obtained their permission. No animals were harmed during collection of fecal samples. The research protocol was reviewed and approved by the Research Ethics Committee of the Northwest A&F University.

### Sample collection

**Figure 1:**
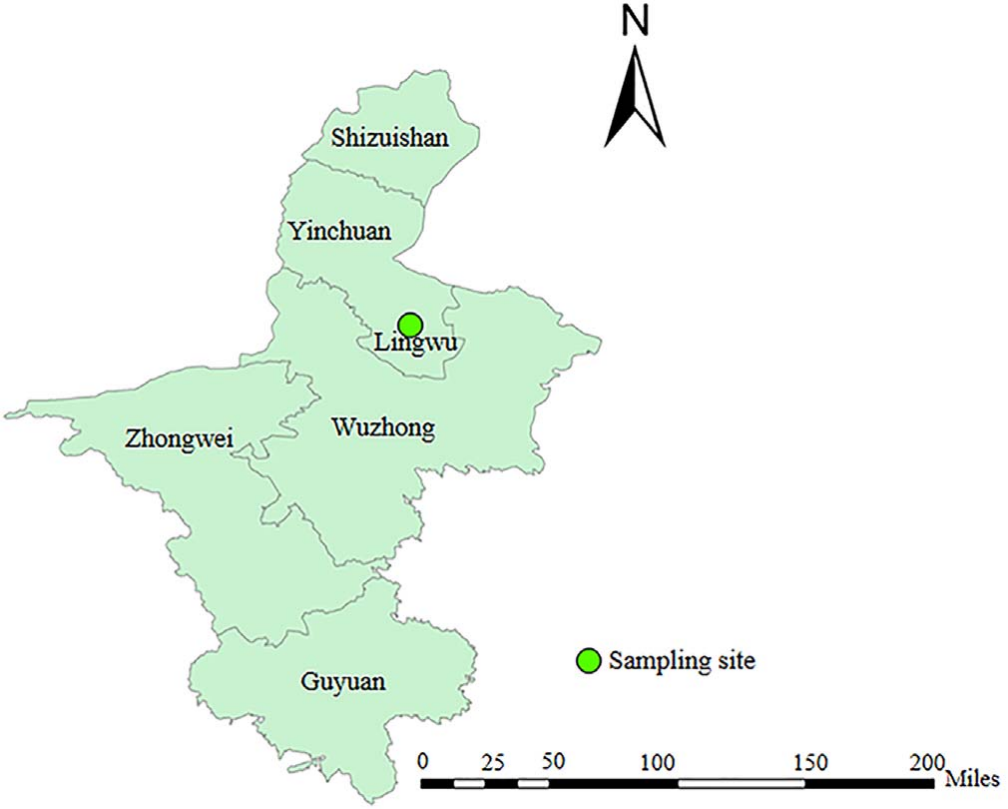
Geographic location of sampling sites in Lingwu County, Ningxia Hui Autonomous Region, China.

From March 2023 to July 2023, a total 343 fecal samples were collected from diarrheic Holstein dairy calves from five large-scale dairy farms in Lingwu County, Ningxia Hui autonomous region, China (Fig. 1). On these farms, newborn dairy calves are raised in separate stalls and weaned at 64 days. The calves are integrated into different pens capable of accommodating 15–20 calves. After six months, they are transferred to the young heifer oxtall for further growth. In this study, we divided the age groups of diarrheic dairy calves into 1–63 and 64–180 days according to pre-weaned and post-weaned calves. In addition, the highest frequency of diarrhea has been reported in calves aged 1–21 days, and calves under 7 days old are more likely to develop severe diarrhea leading to death [4, 8]. Accordingly, we divided pre-weaned calves aged 1–63 days into three age groups (1–7, 8–21, and 22–63 days).

All calves with diarrhea were sampled. Approximately 5 g of fresh fecal samples were collected from the ground using disposable polyethylene gloves, placed into a 15 mL centrifuge tube (Thermo Fisher Scientific, Waltham, MA, USA), clearly marked with the information including farm name, age, and sampling date, and swiftly transported to the laboratory for subsequent DNA extraction under cool conditions.

### DNA extraction

Approximately 200 mg of each fecal sample was used for DNA extraction using an E.Z.N.A. Stool DNA kit (Omega, Norcross, GA, USA). All DNA samples were stored at −40 °C before PCR analysis.

### Investigation on the occurrence and genetic diversity of *Cryptosporidium* spp., *G. duodenalis*, and *E. bieneusi*

A nested PCR-based sequencing tool targeting the *SSU* rRNA gene (~830 bp) was applied to investigate the occurrence of *Cryptosporidium* spp*.* in the fecal samples [34]. Then, a nested PCR-based sequencing tool targeting the *gp60* gene was used to subtype *C. parvum* isolates, as previously reported [57].

Three nested PCR-based sequencing tools targeting the *bg* gene (~511 bp) [37], *tpi* gene (~530 bp) [55], and *gdh* gene (~392 bp) [10] were used to investigate the occurrence and assemblages of *G. duodenalis*, as described previously.

A nested PCR-based sequencing tool targeting the ITS gene (~392 bp) was applied to identify the occurrence and genotypes of *E. bieneusi*, as previously reported [56].

### DNA sequence analysis

For *Cryptosporidium* spp. and *G. duodenalis*, all secondary PCR products were sent to Sangon Biotech (Shanghai, China) for sequencing in both directions using PCR primers in the secondary round. For *E. bieneusi*, due to the high positive rates, only partial positive products were applied for sequence analysis. The sequences obtained were assembled using ChromasPro V1.33 (www.technelysium.com.au/ChromasPro.html), edited with BioEdit V7.04 (www.mbio.ncsu.edu/BioEdit/bioedit.html), and aligned with ClustalX V2.1 (www.clustal.org/).

### Pathogenic bacteria identification

For detection of diarrheagenic *E. coli*, seven PCRs targeting the key virulence determinants of DEC, including *stx1*, *stx2*, *st*, *lt*, *eaeA*, *aggR*, and ipaH, were used [13, 42, 61]. For *C. perfringens*, six PCRs targeting the *cpa*, *cpb*, *etx*, *itx*, *cpe*, and *netB* genes, respectively, were used to detect seven toxinotypes [7, 32]. *Salmonella* spp. were detected using a specific PCR targeting the *invA* gene [47].

### Statistical analysis

Differences in the positive rates of *Cryptosporidium* spp., *G. duodenalis*, *E. bieneusi*, *E. coli*, *C. perfringens*, and *Salmonella* spp. in dairy calves with diarrhea between farms, ages, and seasons were analyzed by using the *χ*^2^ test in SPSS V18.0 (IBM, Armonk, NY, USA). Statistically significant differences were confirmed for a *p* value < 0.05. Moreover, 95% confidence intervals (95% CIs) for the overall positive rates of these zoonotic pathogens were calculated by using SPSS V18.0.

### Nucleotide sequence accession numbers

Representative sequences in the present study were submitted to GenBank under accession numbers PQ008463–PQ008467 and PQ042382–PQ042390 for *SSU* rRNA and the *gp60* gene of *Cryptosporidium* spp., PQ034538–PQ034542, PQ034543–PQ034547, and PQ042375–PQ042381 for the *bg, tpi*, and *gdh* genes of *G. duodenalis*, and PQ032384–PQ032389 for the ITS gene of *E. bieneusi*, respectively.

## Results

### Occurrence of *Cryptosporidium* spp. in dairy calves with diarrhea

PCR analysis showed that 79 out of 343 fecal samples (23.0%; 95% CI, 18.6–27.5) from diarrheic dairy calves were positive for *Cryptosporidium* infection (Table 1). There was a significant difference (*χ*^2^ = 32.007; *df* = 4; *p* < 0.001) in the positive rates of *Cryptosporidium* spp. among the five farms investigated, with positive rates ranging from 10.5% (2/19) to 50.0% (30/60). Significant differences (*χ*^2^ = 22.652; *df* = 3; *p* < 0.001) in the positive rates of *Cryptosporidium* spp. were also found among four age groups. The highest positive rate was found in calves aged 22–63 days (39.1%, 27/69), followed by 8–21 days (27.9%, 34/122), and 64–180 days (12.8%, 14/109). The lowest positive rate was identified in calves aged 1–7 days (9.3%, 4/43). Furthermore, a significant difference (*χ*^2^ = 6.337; *df* = 1; *p* = 0.012) in positive rates of *Cryptosporidium* spp. between the two investigated seasons was observed, with 18.8% (42/223) in spring and 30.8% (37/120) in summer.

**Table 1:** Occurrence and distribution of *Cryptosporidium* species and *C. parvum* subtypes in fecal samples of diarrheic dairy calves from five farms in Lingwu county.

Factor		No. samples	No. positive samples (%)	Species (no.)	*C. parvum* subtype (no.)
Farm					
	Farm 1	60	30 (50.0)	*C. parvum* (21), *C. bovis* (9)	IIdA20G1 (13), IIdA14G1 (1), IIdA15G1 (1), IIdA19G1 (1)
	Farm 2	64	8 (12.5)	*C. parvum* (7), *C. bovis* (1)	IIdA19G1 (3), IIdA20G1 (2), IIdA15G1 (1)
	Farm 3	59	13 (22.0)	*C. parvum* (13)	IIdA15G1 (10), IIdA13G1 (1)
	Farm 4	141	26 (18.4)	*C. parvum* (26)	IIdA15G1 (25)
	Farm 5	19	2 (10.5)	*C. parvum* (2)	IIdA15G1 (2)
Age (days)					
	1–7	43	4 (9.3)	*C. parvum* (4)	IIdA15G1 (2), IIdA20G1 (1)
	8–21	122	34 (27.9)	*C. parvum* (34)	IIdA15G1 (25), IIdA20G1 (7), IIdA19G1 (1)
	22–63	69	27 (39.1)	*C. parvum* (22), *C. bovis* (5)	IIdA15G1 (12), IIdA19G1 (3), IIdA20G1 (3)
	64–180	109	14 (12.8)	*C. parvum* (9), *C. bovis* (5)	IIdA20G1 (4), IIdA13G1 (1), IIdA14G1 (1)
Season					
	Spring	223	42 (18.8)	*C. parvum* (40), *C. bovis* (2)	IIdA15G1 (32), IIdA19G1 (3), IIdA20G1 (2), IIdA14G1 (1)
	Summer	120	37 (30.8)	*C. parvum* (29), *C. bovis* (8)	IIdA20G1 (13), IIdA15G1 (7), IIdA13G1 (1), IIdA19G1 (1)
Total		343	79 (23.0)	*C. parvum* (69), *C. bovis* (10)	IIdA15G1 (39), IIdA20G1 (15), IIdA19G1 (4), IIdA13G1 (1), IIdA14G1 (1)

Sequence analysis of the *SSU* rRNA gene indicated the presence of two *Cryptosporidium* species, namely *C. parvum* (*n* = 69) and *C. bovis* (*n* = 10) (Table 1). Both *C. parvum* and *C. bovis* were detected on Farms 1 and 2, while only *C. parvum* was detected on the other three farms. In the four age groups, *C. parvum* was detected in calves aged 1–7 and 8–21 days, whereas both *C. parvum* and *C. bovis* were detected in dairy calves aged 22–63 and 64–180 days. Additionally, both species were found in calves both in spring and summer.

Further sequence analysis targeting the *gp60* gene indicated five subtypes of *C. parvum* in diarrheic dairy calves in this study (Table 1), with IIdA15G1 (*n* = 39) as the dominant subtype, followed by IIdA20G1 (*n* = 15), IIdA19G1 (*n* = 4), IIdA13G1 (*n* = 1), and IIdA14G1 (*n* = 1). Of these subtypes, IIdA13G1, which lacks a TCA repeat in the trinucleotide repeat region compared to the IIdA14G1 reference sequence (GenBank accession number: MT680897), was identified as a novel subtype of *C. parvum*.

### Occurrence of *G. duodenalis* in dairy calves with diarrhea

The total positive rate of *G. duodenalis* in fecal samples from diarrheic dairy calves was 20.4% (70/343; 95% CI, 16.1–24.7), with positive rates of 15.7% (54/343) for the *bg* gene, 5.2% (18/343) for the *tpi* gene, and 16.6% (57/343) for the *gdh* gene (Table 2). Statistically significant differences (*χ*^2^ = 131.739; *df* = 3; *p* < 0.001) in the positive rates of *G. duodenalis* were found among the four age groups, with the highest 56.9% (62/109) in calves aged 64–180 days and the lowest (0.8%, 1/122) in calves aged 1–7 days. However, no significant differences in the positive rates of *G. duodenalis* were observed across five farms (*χ*^2^ = 3.458; *df* = 4; *p* = 0.484) and two seasons (*χ*^2^ = 0.961; *df* = 1; *p* = 0.327).

**Table 2:** Occurrence of *Giardia duodenalis* assemblages in fecal samples of diarrheic calves from five farms in Lingwu county.

Factor		No. samples	No. positive samples (%)			Assemblage (no. samples)		
			*bg*	*tpi*	*gdh*	*bg*	*tpi*	*gdh*
Farm								
	Farm 1	60	11 (18.3)	4 (6.7)	7 (11.7)	E (11)	E (2), A (2)	E (7)
	Farm 2	64	13 (20.3)	6 (9.4)	15 (23.4)	E (13)	E (6)	E (15)
	Farm 3	59	8 (13.6)	3 (5.1)	8 (13.6)	E (8)	E (3)	E (8)
	Farm 4	141	16 (11.3)	2 (1.4)	21 (14.9)	E (16)	E (2)	E (21)
	Farm 5	19	6 (31.6)	3 (15.8)	6 (31.6)	E (5), A (1)	E (2), A (1)	E (5), A (1)
Age (days)								
	1–7	43	0 (0.0)	0 (0.0)	1 (2.3)	–	–	E (1)
	8–21	122	2 (1.6)	0 (0.0)	0 (0.0)	E (2)	–	–
	22–63	69	5 (7.2)	2 (2.9)	3 (4.3)	E (5)	E (2)	E (3)
	64–180	109	47 (43.1)	16 (14.7)	53 (48.6)	E (46), A (1)	E (13), A (3)	E (52), A (1)
Season								
	Spring	223	37 (16.6)	11 (4.9)	46 (20.6)	E (36), A (1)	E (10), A (1)	E (45), A (1)
	Summer	120	17 (14.2)	7 (5.8)	11 (9.2)	E (17)	E (5), A (2)	E (11)
Total		343	54 (15.7)	18 (5.2)	57 (16.6)	E (53), A (1)	E (15), A (3)	E (56), A (1)

Note: N-dash (-) indicates that no data were obtained.

Further sequence analyses identified the occurrence of assemblages E (*n* = 67), A (*n* = 1), and mixed E and A (*n* = 2) of *G. duodenalis* (Table 2). Assemblages E and mixed E and A were found on Farm 1. Assemblages E and A were found on Farm 5, while only assemblage E was found on the other three farms. Meanwhile, assemblages E, A, and mixed E and A were found in calves aged 64–180 days, but only assemblage E was found in the remaining three age groups. Additionally, assemblages E and A were found in spring, while assemblages E and mixed E and A were found in summer.

### Occurrence of *E. bieneusi* in dairy calves with diarrhea

Of 343 fecal samples from diarrheic calves, 149 (43.4%; 95% CI, 38.2–48.7) were positive for *E. bieneusi* (Table 3). The positive rates (*χ*^2^ = 18.388; *df* = 4; *p* = 0.001) differed significantly among the five farms, with the highest (60%, 36/60) on Farm 1 and the lowest (5.3%, 1/19) on Farm 5. However, no statistically significant differences were found in the positive rates among the four age groups (*χ*^2^ = 5.337; *df* = 3; *p* = 0.149) and two seasons (*χ*^2^ = 0.782; *df* = 1; *p* = 0.377).

**Table 3:** Occurrence of *Enterocytozoon bieneusi* genotypes in fecal samples of diarrheic calves from five farms in Lingwu county.

Factor		No. samples	No. positive samples (%)	No. sequenced samples	Genotype (no. samples)
Farm					
	Farm 1	60	36 (60.0)	19	J (15), EbpC (2), Henan-IV (2)
	Farm 2	64	29 (45.3)	18	J (12), Henan-IV (4), EbpC (1), ESH-01 (1)
	Farm 3	59	24 (40.7)	10	EbpC (6), Henan-IV (3), EbpA(1)
	Farm 4	141	59 (41.8)	29	Henan-IV (12), J (7), EbpC (7), I (3)
	Farm 5	19	1 (5.3)	1	J (1)
Age (days)					
	1–7	43	15 (34.9)	6	Henan-IV (4), Ebpc (1), J (1)
	8–21	122	47 (38.5)	17	Henan-IV (6), EbpC (6), J (4), EbpA (1)
	22–63	69	31 (44.9)	12	Henan-IV (7), EbpC (3), ESH-01 (1), J (1)
	64–180	109	56 (51.4)	42	J (29), EbpC (6), Henan-IV (4), I (3)
Season					
	Spring	223	93 (41.7)	35	J (17), Henan-IV (13), I (3), EbpA (1), ESH-01 (1)
	Summer	120	56 (46.7)	42	J (18), EbpC (16), Henan-IV (8)
Total		343	149 (43.4)	77	J (35), Henan-IV (21), EbpC (16), I (3), EbpA (1), ESH-01 (1)

Six ITS genotypes of *E. bieneusi* were identified among sequenced positive samples, namely J, Henan-IV, EbpC, I, EbpA, and ESH-01 (Table 3). Of them, J was the most frequent genotype observed in 45.5% (35/77) of dairy calves, followed by Henan-IV (27.3%, 21/77), EbpC (20.8%, 16/77), I (3.9%, 3/77), EbpA (1.3%, 1/77), and ESH-01 (1.3%, 1/77). The genotypes of *E. bieneusi* were distributed differently across the farms, with three (J, EbpC, and Henan-IV), four (J, Henan-IV, EbpC, and ESh-01), three (Ebpc, Henan-IV, and EbpA), four (Henan-IV, J, EbpC, and I) and one (J) genotypes on Farms 1–5, respectively. Meanwhile, three (Henan-IV, EbpC, and J), four (Henan-IV, EbpC, J, and EbpA), four (Henan-IV, EbpC, ESH-01, and J) and four (J, EbpC, Henan-IV, and I) genotypes were identified in calves aged 1–7, 8–21, 22–63, and 64–180 days, respectively. In addition, five (J, Henan-IV, I, EbpA, and ESH-01) and three (J, EbpC, and Henan-IV) genotypes were observed in spring and summer, respectively.

**Figure 2:**
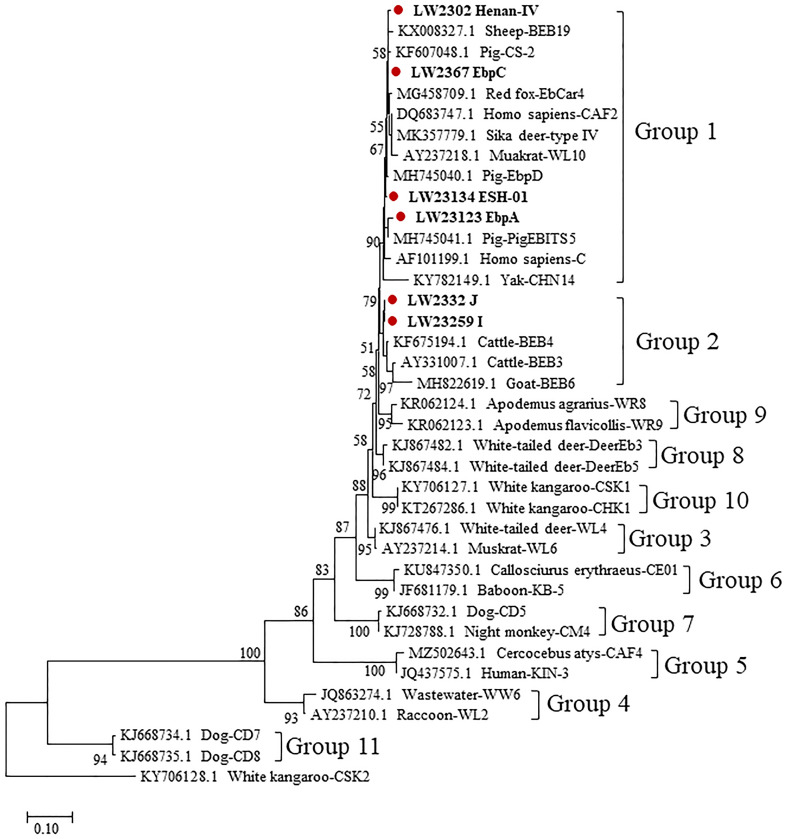
Phylogenetic relationships of representative sequences for the ITS genotypes of *Enterocytozoon bieneusi* identified in this study with reference sequences by Neighbor-joining (NJ) analysis using the Kimura 2-parameter model. Red-filled circles before the bold sample names represent genotypes identified in this study. Bootstrap values of *N* > 50% from 5000 replicates are shown at the nodes. Genotype CSK2 from White kangaroo (KY706128.1) was used as the outgroup.

Phylogenetic analyses based on the ITS gene sequences of *E. bieneusi* showed that all 77 positive samples sequenced were of known genotypes in phylogenetic groups (Fig. 2). The genotypes Henan-IV, Ebpc, EbpA, and ESH-01 belong to Group 1, and have the potential for zoonotic or cross-species transmission. Genotypes J and I belong to Group 2, which also implies public health concerns due to their zoonotic potential.

### Occurrence of *E. coli* in dairy calves with diarrhea

The overall positive rate of DEC in fecal samples from diarrheic dairy calves was 51.9% (178/343; 95% CI, 46.6–57.2) (Table 4). The positive rates of DEC varied significantly (*χ*^2^ = 42.376; *df* = 4; *p* < 0.001) among the farms, with the highest on Farm 5 (94.7%, 18/19) and lowest on Farm 4 (33.3%, 47/141). There were also statistically significant differences (*χ*^2^ = 75.113; *df* = 3; *p* < 0.001) in positive rates among age groups. Higher positive rates were detected in calves aged 64–180 days (79.8%, 87/109) and 22–63 days (63.8%, 44/69) compared to those aged 1–7 days (34.9%, 15/43), and 8–21 days (26.2%, 32/122). Concerning the two seasons examined, a statistically significant (*χ*^2^ = 28.903; *df* = 1; *p* < 0.001) difference was observed in the positive rates of DEC, with the highest rate in summer (71.7%, 86/120).

**Table 4:** Occurrence of diarrheagenic *Escherichia coli* virulence genes in fecal samples of diarrheic calves from five farms in Lingwu county.

Factor		No. samples	No. positive samples (%)	Virulence genes (no. samples)						
				*stx1*	*stx2*	*st*	*lt*	*eaeA*	*aggR*	*ipaH*
Farm										
	Farm 1	60	41 (68.3)	21	20	20	0	26	0	0
	Farm 2	64	36 (56.3)	18	16	2	0	25	0	0
	Farm 3	59	36 (61.0)	23	15	5	0	25	0	0
	Farm 4	141	47 (33.3)	35	20	4	0	30	0	0
	Farm 5	19	18 (94.7)	12	12	4	0	13	0	0
Age (days)										
	1–7	43	15 (34.9)	5	5	4	0	9	0	0
	8–21	122	32 (26.2)	19	5	11	0	13	0	0
	22–63	69	44 (63.8)	23	11	20	0	30	0	0
	64–180	109	87 (79.8)	62	62	0	0	67	0	0
Season										
	Spring	223	92 (41.3)	59	39	11	0	61	0	0
	Summer	120	86 (71.7)	50	44	24	0	58	0	0
Total		343	178 (51.9)	109	83	35	0	119	0	0

PCR analysis showed that four (*eaeA*, *stx1*, *stx2,* and *st*) of seven virulence genes of diarrheagenic *E. coli* were detected in diarrheic dairy calves, with *eaeA* (*n* = 119) being the dominant one, followed by *stx1* (*n* = 109), *stx2* (*n* = 83), and *st* (*n* = 35) (Table 4). Of these, 62 (18.1%), 66 (19.2%), 48 (14.0), and 2 (0.6%) fecal samples were positive for one, two, three, and four virulence genes, respectively.

### Occurrence of *C. perfringens* in dairy calves with diarrhea

Of 343 fecal samples from diarrheic dairy calves, 211 (61.5%; 95% CI, 56.3–66.7) were positive for *C. perfringens* (Table 5). There were statistically significant differences (*χ*^2^ = 13.169; *df* = 4; *p =* 0.010) in the positive rates of *C. perfringens* among farms. The highest positive rate of *C. perfringens* was found in calves on Farm 3 (78.0%, 46/59), followed by Farm 5 (73.7%, 14/19), Farm 4 (61.0%, 86/141), Farm 1 (56.7%, 34/60), and Farm 2 (48.4%, 31/64)*.* There were also significant differences (*χ*^2^ = 26.637; *df* = 1; *p* < 0.001) in the positive rates of *C. perfringens* between the two seasons, with a higher positive rate of *C. perfringens* in calves in summer (80.0%, 96/120) compared to spring (51.6%, 115/223). However, no significant differences (*χ*^2^ = 0.960; *df* = 3; *p* = 0.811) were found in the positive rates of *C. perfringens* among the four age groups. Analysis of toxin genes revealed the presence of only the *cpa* gene (*n* = 211) among *C. perfringens* positive samples. The remaining toxin genes investigated (*cpb*, *etx*, *itx*, *cpe*, and *netB*) were not detected in this study (Table 5).

**Table 5:** Occurrence of *Clostridium perfringens* toxinotypes in fecal samples of diarrheic calves from five farms in Lingwu county.

Factor		No. samples	No. positive samples (%)	Toxin gene (no. samples)					
				*cpa*	*cpb*	*etx*	*itx*	*cpe*	*netB*
Farm									
	Farm 1	60	34 (56.7)	34	0	0	0	0	0
	Farm 2	64	31 (48.4)	31	0	0	0	0	0
	Farm 3	59	46 (78.0)	46	0	0	0	0	0
	Farm 4	141	86 (61.0)	86	0	0	0	0	0
	Farm 5	19	14 (73.7)	14	0	0	0	0	0
Age (days)									
	1–7	43	29 (67.4)	29	0	0	0	0	0
	8–21	122	76 (62.3)	76	0	0	0	0	0
	22–63	69	41 (59.4)	41	0	0	0	0	0
	64–180	109	65 (59.6)	65	0	0	0	0	0
Season									
	Spring	223	115 (51.6)	115	0	0	0	0	0
	Summer	120	96 (80.0)	96	0	0	0	0	0
Total		343	211 (61.5)	211	0	0	0	0	0

### Co-infections

**Figure 3:**
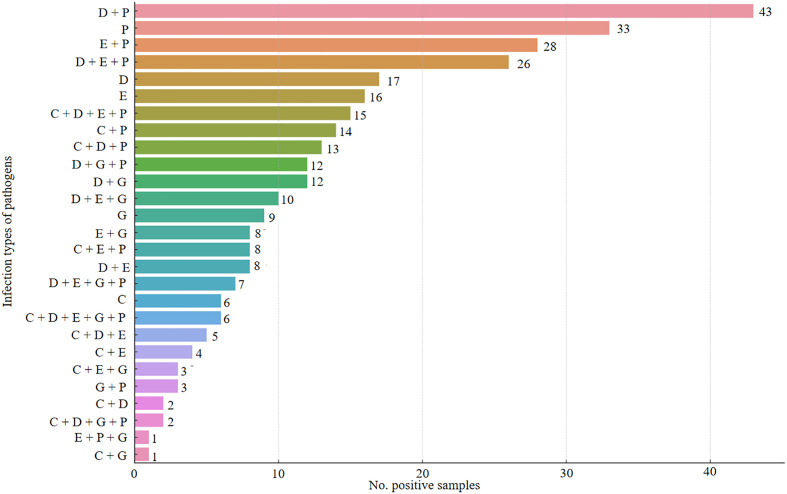
Infection types of pathogens in fecal samples of diarrheic calves from five farms in Lingwu county. C/D/E/G/P represent *Cryptosporidium* spp., diarrheagenic *Escherichia coli*, *Enterocytozoon bieneusi*, *Giardia duodenalis*, and *Clostridium perfringens*, respectively.

Among the 343 fecal samples from diarrheic dairy calves examined, five (*Cryptosporidium* spp., *Giardia duodenalis*, *Enterocytozoon bieneusi*, *E. coli*, and *C. perfringens*) of six pathogens were detected in the positive samples, with an overall positive rate of 91.0% (312/343; 95% CI, 87.9–94.0). In all, 231 (67.3%; 95% CI, 62.4–72.3) positive samples were found to be co-infections, with 123 (35.9%), 78 (22.7%), 24 (7.0%) and 6 (1.7%) samples positive for two, three, four, and five pathogens, respectively. The most common type was co-infection with DEC and *C. perfringens* (*n* = 43) (Fig. 3). Of co-infections with two pathogens, a total of ten types were identified, with co-infections of *C. perfringens* and DEC being the most frequent (*n* = 43). Concerning co-infections of three pathogens, eight types were found, with co-infections of *C. perfringens*, DEC, and *E. bieneusi* (*n* = 26) being the dominant one. Concerning co-infections of four pathogens, three types were found, with co-infections of *C. perfringens*, DEC, *E. bieneusi*, and *Cryptosporidium* spp. (*n* = 15) being the most frequent.

## Discussion

In this study, the overall positive rate of *Cryptosporidium* spp. in diarrheic dairy calves from five farms in Lingwu County, Ningxia Hui Autonomous Region was 23.0% (79/343), which was significantly higher than that (4.2%, 59/1414) observed in dairy cattle in Gansu Province, and that (5.5%, 92/1688) in Ningxia Hui Autonomous Region [66, 73], similar to that (24.0%, 93/388) in pre-weaned dairy calves in Guangdong Province, and that (26.1%, 43/165) in dairy cattle in Jiangxi Province [22, 39], but lower than that (37.7%, 55/146) in diarrheic dairy cattle in Central Inner Mongolia Autonomous Region, and that (47.7%, 72/151) in pre-weaned dairy calves in Heilongjiang Province [72, 74]. These disparities in the positive rates of *Cryptosporidium* infection further confirmed that the positive rates of *Cryptosporidium* infection may be related to geographic location, age, and animal health status. The higher positive rate of *Cryptosporidium* infection observed in this study, compared to those previously reported in northwest China, may be attributed to the fact that all samples were collected from diarrheic dairy calves. Additionally, feeding management, sample sizes, animal species, and breeds can also influence the positive rate.

The positive rates of *Cryptosporidium* varied significantly among the age groups and were significantly higher in 8–21 day and 22–63 day pre-weaned calves than in 64–180 day post-weaned calves. Of note, Gong et al. found that the highest infection rate was observed in pre-weaned calves in China [27]. However, a lower positive rate was also observed in newborn calves aged 1–7 days, which may be due to the immunoprotective properties of colostrum [9]. Meanwhile, the positive rate of *Cryptosporidium* in diarrheic dairy calves was significantly higher in summer compared to spring, which was in accordance with previous studies conducted in Xinjiang and Henan Province [64, 70]. The increase in positive rates of *Cryptosporidium* in summer is likely to be the result of accumulative infection caused by animals [43].

Sequence analysis revealed the presence of two *Cryptosporidium* species (*C. parvum* and *C. bovis*) in diarrheic dairy calves. *Cryptosporidium parvum*, a zoonotic species commonly found in humans and various animals, was the main species infecting pre-weaned calves. While *C. bovis* mainly infected post-weaned calves [52]. In our study, we noticed an interesting phenomenon whereby *C. parvum* was detected only in calves aged 1–7 and 8–21 days. Both *C. parvum* and *C. bovis* were detected in 22–63 and 64–180 day-old calves, and the proportion of *C. bovis* gradually increased with age. The reason for this phenomenon may be related to the different timing of peak oocyst shedding in *C. parvum* and *C. bovis*, with *C. parvum* peaking in the second week, while *C. bovis* peaks in the sixth week, as reported in a longitudinal survey of calves [31].

Further subtyping analysis identified five subtypes in *C. parvum*-positive samples, namely IIdA15G1, IIdA20G1, IIdA19G1, IIdA13G1, and IIdA14G1. Among these, IIdA15G1 was the dominant one, followed by IIdA20G1, consistent with previous reports in northwestern and southern China [29]. The remaining subtypes, IIdA19G1, IIdA14G1, and the higher genetic diversity of *C. parvum* subtypes, have also been reported in Xinjiang, China [68]. Notably, IIdA13G1 identified in this study was first reported in *C. parvum.* The emergence of new subtypes may be a process of pathogen-host interaction [12].

The positive rate of *G. duodenalis* detected in this research was 20.4% (70/343), which was similar to that (19.9%, 31/156) reported in cattle in eastern Taiwan [38], higher than that (9.3%, 10/108) observed in beef cattle in southwest Inner Mongolia [26], but lower than that (27.5%, 144/524) in dairy cattle in Yunnan and that (74.2%, 288/388) in pre-weaned dairy calves in Guangdong [22, 30]. In our study, the positive rate of *G. duodenalis* was significantly higher in post-weaned calves aged 64–180 days than in pre-weaned calves aged <64 days, which was consistent with a previous study in central Inner Mongolia in northern China, but the opposite results were obtained in Henan, Yunnan, and Xinjiang, where the positive rates were higher in pre-weaned calves [30, 63, 75, 76].

In our study, assemblage E was dominant in 67 of the 70 *G. duodenalis-*positive samples. This assemblage has been commonly detected in previous studies of dairy calves from other regions in China, including Hubei, Henan, and Xinjiang [21, 46, 63]. Assemblage E was previously thought to primarily infect hoofed mammals, but the presence of assemblage E was found in humans in recent studies, indicating that this assemblage has zoonotic potential [11, 51]. In addition, assemblage A and mixed E and A were found in this study. Assemblage A, a zoonotic assemblage, was a leading cause of giardiasis in humans, which can infect humans and various animals [24]. Therefore, these results emphasized the importance of public health implications of *G. duodenalis* in this study.

Sequence analysis of the ITS locus from 77 isolates of *E. bieneusi* identified six known genotypes (J, Henan-IV, Ebpc, I, EbpA, and ESH-01). Among them, genotypes J and I belong to Group 2, which is prevalent in dairy cattle worldwide. Although this group was previously reported to be ruminant-adapted, public health concerns have arisen with the discovery of I, J and other genotypes in this group, such as BEB4 and BEB6 in humans and various animals [40, 54]. The remaining genotypes, Henan-IV, EbpC, EbpA, and ESH-01 belong to Group 1. The majority of the genotypes in this group are zoonotic and potentially pose a threat to humans and animals [40].

Of the 343 diarrheic fecal samples tested by PCR in this experiment, 51.9% (178/343) carried at least one or more examined virulence genes. Overall, DEC positive rates were lower than that (79.0%, 79/100) found in cattle in Hyogo Prefecture, Japan, and that (77.0%, 77/100) in diarrheic dairy calves in the Nile Delta reported in previous studies [2, 6]. Geography, sampling methods, and environmental disinfection practices on farms may account for these differences. PCR amplification of virulence genes showed the presence of *eaeA*, *stx1*, *stx2*, and *st*, with *eaeA* being the dominant one. However, no *lt*, *aggR* or *ipaH* virulence genes were detected in the fecal samples examined in this study. In addition, multiple key virulence factors for DEC were detected in 116 of 343 fecal samples, with *eaeA*, *stx1*, and *stx2* predominating. These multiple virulence factors are associated with hemorrhagic colitis (HC) and bloody diarrhea in humans, causing a serious risk of zoonotic disease [19, 45].

The positive rate of *C. perfringens* in fecal samples from diarrheic dairy calves was 61.5% (211/343), which was higher than that (49.6%, 56/113) in Xinjiang, from abattoirs (21.2%, 150/708) in Shaanxi, and fresh beef samples from supermarkets in Beijing (24.0%, 53/221) [33, 35, 65]. In these studies, *C. perfringens* isolates were identified as toxinotypes A and D, and type A was predominant. However, in our survey, only the *cpa* gene was detected as positive, thus all *C. perfringens* isolates were type A. CPA toxin, as the only major toxin of *C. perfringens* type A, has been showed to be essential for intestinal virulence in a calf intestinal loop model [28]. At the same time, CPA is the most important virulence factor causing gas gangrene or histotoxic infections in humans [5, 25, 62]. These findings highlighted the serious threat of *C. perfringens* type A both in calves and humans.

In this study, 91.0% of diarrheic calves were positive for at least one of the examined enteric pathogens, indicating that the infectious factor remains a significant contributor to calf diarrhea in Lingwu county, Ningxia Hui Autonomous Region. Notably, 67.3% of the calves had mixed infections of two or more pathogens. It is difficult to determine the specific role of each pathogen in calves with diarrhea, as they could produce a similar clinic syndrome in isolation or association [18]. Furthermore, co-infections with multiple enteric pathogens also appears to exacerbate the severity of diarrhea [71]. Therefore, measures such as disinfecting environmental sanitation and reducing stocking density are needed to prevent and control the occurrence of calf diarrhea.

## Conclusion

The present study explored the occurrence and genetic diversity of *Cryptosporidium* spp., *G. duodenalis*, *E. bieneusi*, *E. coli*, and *C. perfringens* from diarrheic dairy calves in Lingwu county, Ningxia Hui Autonomous Region. The results of this study indicate high positive rates and zoonotic potential of these five pathogens. Meanwhile, the positive rates and genetic diversity identified were related to locations, ages, and seasons. Considering the zoonotic potential of these pathogens, interventions are urgently needed to reduce the possibility of cross-species transmission between calves and humans.
